# The C-terminal helix in the YjeQ zinc-finger domain catalyzes the release of RbfA during 30S ribosome subunit assembly

**DOI:** 10.1261/rna.049171.114

**Published:** 2015-06

**Authors:** Ajitha Jeganathan, Aida Razi, Brett Thurlow, Joaquin Ortega

**Affiliations:** Department of Biochemistry and Biomedical Sciences, M.G. DeGroote Institute for Infectious Diseases Research, McMaster University, Hamilton, Ontario, Canada L8S 4K1

**Keywords:** ribosome assembly, 30S subunit, YjeQ protein, RsgA protein, RbfA protein, GTPase

## Abstract

YjeQ (also called RsgA) and RbfA proteins in *Escherichia coli* bind to immature 30S ribosome subunits at late stages of assembly to assist folding of the decoding center. A key step for the subunit to enter the pool of actively translating ribosomes is the release of these factors. YjeQ promotes dissociation of RbfA during the final stages of maturation; however, the mechanism implementing this functional interplay has not been elucidated. YjeQ features an amino-terminal oligonucleotide/oligosaccharide binding domain, a central GTPase module and a carboxy-terminal zinc-finger domain. We found that the zinc-finger domain is comprised of two functional motifs: the region coordinating the zinc ion and a carboxy-terminal α-helix. The first motif is essential for the anchoring of YjeQ to the 30S subunit and the carboxy-terminal α-helix facilitates the removal of RbfA once the 30S subunit reaches the mature state. Furthermore, the ability of the mature 30S subunit to stimulate YjeQ GTPase activity also depends on the carboxy-terminal α-helix. Our data are consistent with a model in which YjeQ uses this carboxy-terminal α-helix as a sensor to gauge the conformation of helix 44, an essential motif of the decoding center. According to this model, the mature conformation of helix 44 is sensed by the carboxy-terminal α-helix, which in turn stimulates the YjeQ GTPase activity. Hydrolysis of GTP is believed to assist the release of YjeQ from the mature 30S subunit through a still uncharacterized mechanism. These results identify the structural determinants in YjeQ that implement the functional interplay with RbfA.

## INTRODUCTION

The 70S ribosome is the macromolecular complex performing protein synthesis. It is comprised of two functional subunits, the large 50S and the small 30S subunits (Ramakrishnan 2002). The 50S subunit is composed of the 23S and 5S rRNAs as well as 34 ribosomal proteins (r-proteins) (Ban et al. 2000; Harms et al. 2001). The 50S subunit houses the peptidyl transferase center (PTC), which is the catalytic site for peptide bond formation. The 30S subunit contains only one molecule of rRNA (16S rRNA) and 21 r-proteins (Wimberly et al. 2000). The functional core in the 30S subunit is the decoding center, which translates the sequence of the mRNA into protein. Considering the size and complexity of the ribosome, it is a remarkable feat that assembly of the ribosomal subunits in bacterial cells occurs with such high precision and efficiency.

A constant challenge for the cell to maintain high efficiency in the process of ribosome assembly is to ensure that the rRNA molecules stay in a productive line of folding toward the mature structure, without falling into kinetic traps (Woodson 2008, 2011; Shajani et al. 2011). In particular, the folding events involving the maturation of the decoding center in the 30S subunit and PTC in the 50S subunit, occurring at the late stages of assembly (Jomaa et al. 2011a, 2014; Guo et al. 2013; Leong et al. 2013; Li et al. 2013) have a strong tendency to fall into local energy minima. Therefore, bacterial cells have acquired a number of protein factors that are dedicated to assist the folding of these motifs critical for ribosome function (Wilson and Nierhaus 2007); however, it is still not understood how they perform their function.

There are at least four protein factors (YjeQ [or RsgA], RbfA, RimM, and Era) that assist the folding of the decoding center during the late stages of assembly of the 30S subunit. Their roles in the maturation of the functional core of the 30S subunit may entail facilitating proper 17S rRNA folding, assisting processing of the rRNA, or mediating protein–RNA interactions. Cryo-electron microscopy (cryo-EM) (Sharma et al. 2005; Datta et al. 2007; Guo et al. 2011; Jomaa et al. 2011b) revealed that at least three factors, Era, YjeQ, and RbfA bind at or in close proximity to the decoding center at sites that are not overlapping, indicating that simultaneous binding is stereochemically possible. Consistently, genetic experiments (Bylund et al. 1998, 2001; Inoue et al. 2003, 2006; Campbell and Brown 2008) suggested that these factors operate in conjunction rather than independently. Indeed, a recent study (Goto et al. 2011) described that binding of YjeQ stimulates the removal of RbfA bound to the mature 30S subunit. The RbfA protein binds to the small ribosomal subunit at the junction between the body and head, most likely when the particle is still in an immature state and alters the position of helix 44 and 45 (Datta et al. 2007). This study (Goto et al. 2011) suggested that one of the functions of YjeQ is assisting the release of RbfA and perhaps other assembly factors, once maturation of the ribosomal particle is completed. Therefore, accumulated evidence suggests that YjeQ, RbfA, RimM, and Era function together to ensure the maturation of the functional core of the 30S subunit.

Although the question here at large is how these four factors cooperate to ensure proper maturation of the decoding center, the specific focus in this study is to identify the motifs in YjeQ that implement the functional interplay between YjeQ and RbfA (Goto et al. 2011).

The YjeQ protein is a GTPase broadly conserved across most bacterial species. It exhibits slow intrinsic GTPase activity; however, interaction with the 30S subunit enhances this activity by 160-fold (Daigle and Brown 2004). YjeQ is structurally well conserved (Levdikov et al. 2004; Shin et al. 2004; Nichols et al. 2007) featuring an amino-terminal oligonucleotide/oligosaccharide binding (OB-fold) domain, a central GTPase module and a carboxy-terminal zinc-finger domain (Supplemental Fig. S1A). The OB-fold domain consists of antiparallel β-sheets that come together forming a β-barrel. In the YjeQ GTPase domain the characteristic G motifs mediating the nucleotide binding (G1 (Walker A, P-loop)-G2 (T)-G3 (Walker B)-G4 (N/TKxD)-G5 [(T/G)(C/S)A]) are circularly permutated and adopt a G4–G5–G1–G2–G3 pattern. Work in the prototype ras GTPase showed that nucleotide binding and hydrolysis leads to conformational changes typically confined to two loops in the GTPase domain known as switch I and switch II (Hall et al. 2002). Switch I in YjeQ encompass the G2-loop and it is disordered in the YjeQ structures. The G3 loop constitutes the switch II, which in this case is a long stretch of amino acids connecting the GTPase domain with the carboxy-terminal zinc-finger domain. Therefore, switch I and II are well positioned in YjeQ to propagate the conformational changes occurring as a result of the GTP hydrolysis to the upstream OB-fold and downstream zinc-finger domain, respectively. The carboxy-terminal zinc-finger domain in YjeQ is comprised of a 3^10^-helix and a long loop containing three cysteine residues and a histidine that mediate the tetrahedral coordination of one zinc ion. Beyond this loop there are two additional α-helices. The function of the last carboxy-terminal α-helix is unclear, as it is not directly required to coordinate the zinc ion (Supplemental Fig. S1A).

It has been described that the OB-fold domain is essential for binding to the 30S subunit and GTPase stimulation (Daigle and Brown 2004), but a specific role for the GTPase and zinc-finger domains has not been assigned. Here, we found that the zinc-finger domain is comprised of two functional motifs. The region coordinating the zinc ion, which is essential for efficient binding of YjeQ to the 30S subunit and the carboxy-terminal α-helix that is necessary for the removal of RbfA from the mature 30S subunit. In addition, it has been described (Goto et al. 2011) that when the 30S subunit reaches the mature state, the GTPase activity of the bound YjeQ increases. We found that the ability of the mature 30S subunit to stimulate YjeQ GTPase activity also depends on the presence of the carboxy-terminal α-helix in the zinc-finger domain. Our data are consistent with a model in which YjeQ uses the carboxy-terminal α-helix as a sensor to gauge the conformation of helix 44, an essential motif of the decoding center that only adopts its mature conformation at the very end of the assembly process. According to this model, the carboxy-terminal α-helix senses that helix 44 has reached the mature conformation and triggers GTP hydrolysis facilitating the release of YjeQ from the mature 30S subunit through a still uncharacterized mechanism. Release of the assembly factors is necessary for the mature 30S subunit to associate with the 50S subunit and engage in translation.

## RESULTS

### The carboxy-terminal zinc-finger domain of YjeQ provides structural stability to the protein

To determine the specific function of the zinc-finger domain of YjeQ in assisting the assembly of the 30S subunit, we constructed three protein variants with different truncations in the carboxy-terminal region (Supplemental Fig. S1B). In the first variant (YjeQ M1), the entire zinc-finger domain was removed by introducing a stop codon in the position of Leu 278 (*Escherichia coli* numbering) located in the loop connecting this domain with the GTPase domain. For the second variant the stop codon was introduced in the position of Phe 287, right after the 3^10^-helix, therefore removing the loop coordinating the zinc ion. Finally, for the third variant (YjeQ M3) the stop codon was introduced in the position of Lys 320 removing the carboxy-terminal α-helix, but not the loop coordinating the zinc ion.

To compare the stability of these YjeQ variants with the wild-type YjeQ protein, we performed a precipitation test in which the proteins were incubated at different temperatures and NH_4_Cl concentrations (Supplemental Fig. S1C). From these experiments we concluded that in order to maintain the YjeQ variants in solution under the low salt conditions required for our binding assays with the 30S ribosome subunits (see below), the temperature had to be maintained at 16°C or lower.

In addition, to ensure that these carboxy-terminal deletions did not produce a global unfolding of the protein, the wild-type YjeQ protein and YjeQ variants were analyzed by circular dichroism (CD) (Supplemental Fig. S2). This technique, consistent with the results from the precipitation test, showed that at a temperature of 25°C or lower the YjeQ variants were stable and properly folded. However, the carboxy-terminal zinc-finger domain of the protein was necessary for the YjeQ protein to remain structurally stable at higher temperatures.

Finally, we tested the effect of removing the zinc ion on the stability of YjeQ. Three cysteine residues (Cys 297, Cys 302, and Cys 310) and a histidine (His 305) mediate the tetrahedral coordination of one zinc ion to the carboxy-terminal domain of YjeQ (Supplemental Fig. S1A). To remove the ability of the domain to coordinate the zinc ion, Cys 297 and Cys 310 residues were mutated to alanine (YjeQ M5). We found that when expressing this variant most of the protein produced was insoluble regardless of the temperature used in the incubation of the culture (16°C, 25°C, and 37°C) (data not shown). This result suggested that in the full-length YjeQ, the presence of the zinc ion is important not only for the folding of the zinc-finger domain, but also for the proper folding of the amino-terminal OB fold and GTPase domains.

### YjeQ requires the carboxy-terminal zinc-finger domain to bind the mature 30S subunit

**FIGURE 1.**
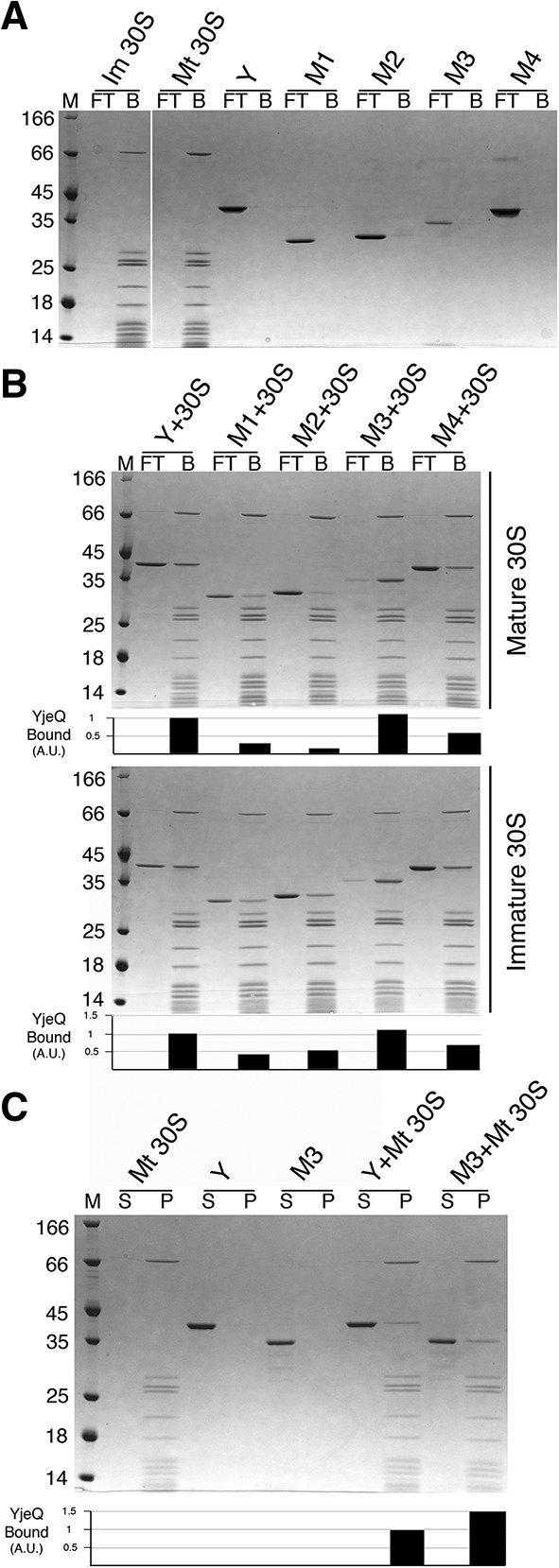
Binding of YjeQ carboxy-terminal variants to the mature and immature 30S subunits. (*A*,*B*) Ability of YjeQ (Y) and YjeQ variants (M1, M2, M3, and M4) to bind to the mature (Mt) and immature (Im) 30S subunits analyzed by filtration assays. Coomassie blue stained SDS-PAGE in *A* contains the controls for the experiment consistent in reactions containing either YjeQ (full-length or variants) or 30S subunits (mature or immature) by themselves. Reactions containing a mixture of YjeQ protein and ribosomal particles contained a fivefold molar excess of protein. Assembly mixtures were incubated for 15 min at 16°C in the presence of 1 mM GMP-PNP. Following incubation, the reactions were passed through a 100 kDa cut-off filter using centrifugation. The unbound protein was captured in the flow-through (FT) and the protein bound to the ribosomal particles (B) was retained by the filter and resuspended by an equal volume of buffer. The molecular weight marker (M) is in kDa. The flow-through and bound portions from these assays were loaded into 4%–12% bis–tris polyacrylamide gels and resolved using SDS-PAGE. (*C*) Pelleting assay of YjeQ M3 variant with the mature 30S subunit. A fivefold excess of YjeQ M3 was incubated with mature 30S subunits for 15 min at 16°C. Following the incubation, reactions were laid over a sucrose cushion and subjected to ultracentrifugation. Proteins that were unbound were collected in the supernatant (S), while proteins that bound to the 30S particle were found in the pellet (P). The molecular weight (M) is in kDa. The pellet and supernatant were resolved by 4%–12% bis–tris SDS-PAGE and stained with Coomassie blue. The bar diagrams *under* the gels in *B* and *C* indicate the binding of the YjeQ variants to the 30S subunits with respect to wild-type YjeQ (set as 1).

The OB-fold domain of YjeQ has already been identified as a necessary motif for the binding of the protein to the 30S subunits and stimulation of its GTPase activity (Daigle and Brown 2004). Here, to determine the role of the zinc-finger domain of YjeQ in the binding of the protein to the small ribosome subunit, we tested the ability of the carboxy-terminal truncation variants of YjeQ (Supplemental Fig. S1B) to bind mature 30S and immature 30S subunits purified from *yjeQ* null *E. coli* cells. To this end, we used filtration assays in which a mixture of the 30S subunits (mature or immature) with YjeQ (full-length or variants) was incubated at 16°C for 15 min. After the incubation period, reactions were spun through a 100 kDa centrifugal device that retains the YjeQ protein or its variants only when bound to the ribosomal particles. The unbound fraction of protein was captured in the flow-through. However, the protein bound to the 30S subunits was retained by the filter and subsequently resuspended. Resolving these samples by SDS-PAGE allowed us to visualize the content of the flow-through and bound fractions (Fig. 1).

In this assay, one of the factors affecting the amount of binding of YjeQ to the 30S subunits is the salt concentration in the reaction buffer. Filtration assays (Supplemental Fig. S3; left panel) performed with buffers containing a concentration of NH_4_Cl ranging from 60 to 600 mM showed that at 300 mM NH_4_Cl, YjeQ bound to the 30S subunit at ∼1:1 ratio, which is the stoichiometry that has been previously established for the 30S+YjeQ complex (Daigle and Brown 2004; Himeno et al. 2004; Guo et al. 2011; Jomaa et al. 2011b). Therefore, we used buffer containing 300 mM NH_4_Cl for these filtration assays, as these conditions were optimal to measure specific binding of YjeQ to the 30S subunits and minimize any potential nonspecific binding. Under these conditions the filter did not retain YjeQ or the YjeQ protein variants when they were by themselves, thus they all appeared in the flow through upon centrifugation. Instead, the filter retained all the mature and immature 30S subunits (Fig. 1A).

Under these conditions, we first tested the ability of full-length YjeQ to bind the mature and immature 30S subunits and found that the protein bound equally well to both particles in the presence of GMP-PNP (Fig. 1B). Then, we tested the binding of the YjeQ variants to the mature 30S subunit (Fig. 1B, top panel). We found that the YjeQ M1 variant lacking the entire zinc-finger domain showed a fourfold decrease in binding to the mature 30S subunit compared with wild-type YjeQ. Similarly, the YjeQ M2 variant, containing only the first 3^10^-helix of the zinc-finger domain, showed a fivefold decrease in binding. However, the YjeQ M3 variant that had only missing the carboxy-terminal α-helix motif showed slightly improved binding compared with wild-type YjeQ (Fig. 1B, top panel).

A caveat in the filtration assay was that not all of YjeQ M3 protein was recovered in the flow-through nor in the bound fractions. This suggested that a percentage of the YjeQ M3 protein was nonspecifically binding to the filter, despite stringent blocking of the filter with solution of bovine serum albumin (BSA). To confirm the ability of the YjeQ M3 variant to bind the mature 30S subunit a pelleting assay was performed with both proteins (Fig. 1C). In the pelleting assay, the binding reaction was laid over a sucrose cushion in the same buffer conditions used for the filtration assay and then subjected to ultracentrifugation. Protein binding to the 30S subunit was found in the pellet fraction and the unbound protein was found in the supernatant fraction. Proteins in both fractions were then visualized by SDS-PAGE. Consistent with the filtration assay (Fig. 1C), the pelleting assay showed that the binding of YjeQ M3 to the mature 30S subunit was ∼1.5-fold enhanced compared to that of wild-type YjeQ (Fig. 1C). Considering that the stoichiometry of the 30S+YjeQ complex is 1:1, these results suggest that under the salt concentration used in the pelleting experiment (300 mM NH_4_Cl) approximately one-third of the YjeQ M3 variant was binding the mature 30S subunit in an unspecific manner. It is plausible that exposure of a new surface in the zinc-finger domain of YjeQ upon removal of the carboxy-terminal α-helix may be causing the unspecific binding.

When analyzing the binding of the YjeQ variants to the immature subunit, the binding of the YjeQ M1 and YjeQ M2 variants only decreased by approximately twofold when compared with wild-type YjeQ. In the case of YjeQ M3, it bound to the immature 30S subunit similarly to wild-type YjeQ (Fig. 1B, bottom panel).

Overall, this data suggest that the zinc-finger domain plays an important role for efficient binding of YjeQ to the mature 30S subunit, but does not require the carboxy-terminal α-helix for this function. It also suggests that the zinc-finger domain may not play as critical as a role for binding to the immature 30S subunit. In this case, efficient binding to the subunit seems to depend more on the OB-fold domain.

### Lysine 298 and arginine 300 in the carboxy-terminal zinc-finger domain of YjeQ are not essential for binding to the mature 30S subunit

A previous structural study describing the cryo-EM structure of the mature 30S subunit in complex with YjeQ (Guo et al. 2011) indicates that YjeQ binds in an orientation in which the OB-fold domain interacts with helix 44 and the zinc-finger domain interacts with the head of the 30S subunit mainly through residues Lys 298 and Arg 300 (Fig. 5A, below). The electrostatic interactions between the positively charged side chains of these two amino acids and the negatively charged phosphate groups of the 16S rRNA of the ribosomal particle stabilize these contacts. To study the contribution of Lys 298 and Arg 300 for binding of YjeQ to the ribosome subunit, we mutated both residues to alanine in the full-length YjeQ protein to produce the YjeQ M4 variant (Supplemental Fig. S1B). In the precipitation test this mutant did not show any instability when exposed at 37°C (Supplemental Fig. S1C) and its CD spectra at all temperatures tested completely overlapped with that of wild-type YjeQ (Supplemental Fig. S2) indicating that the YjeQ M4 variant folds into its native conformation. When we tested the ability of the YjeQ M4 variant to bind the mature and immature 30S subunit (Fig. 1A,B), we found that the YjeQ M4 variant had only decreased binding to the mature and immature 30S subunits by twofold and 1.3-fold, respectively. This result indicated, contrary to what the cryo-EM structure suggested (Guo et al. 2011), that residues Lys 298 and Arg 300 in the zinc-finger domain of YjeQ are not essential for binding to the mature 30S subunit.

### The carboxy-terminal α-helix of the zinc-finger domain is necessary for the 30S subunit-dependent GTPase activity of YjeQ

In the absence of ribosome subunits YjeQ hydrolyzes GTP slowly and exhibits a *k*_cat_ of 9.4 h^−1^ and a *K*_m_ for GTP of 120 μM (Daigle et al. 2002). However, association with the 30S subunit results in a 160-fold stimulation of YjeQ GTPase activity. Previous studies indicated that the first 20 amino-terminal amino acids are important for ribosome-stimulated GTPase activity of YjeQ (Daigle and Brown 2004).

Here, we tested the carboxy-terminal truncations of YjeQ to determine whether the zinc-finger domain plays any role in this activity. The initial rates of GTP hydrolysis for full-length YjeQ and YjeQ variants were measured in the presence and absence of mature and immature 30S subunits. To this end we used a malachite green assay to measure the amount of free phosphate produced in the reaction from GTP hydrolysis. Reactions were incubated for 1 h at 25°C before they were quenched. This temperature was chosen instead of the 30°C that was used in previous studies (Daigle and Brown 2004) with YjeQ, to ensure that the YjeQ variants remained stable during the course of the reaction (Supplemental Figs. S1C, S2). The initial rate of the GTP hydrolysis was estimated from the amount of free phosphate produced in each reaction.

**FIGURE 2.**
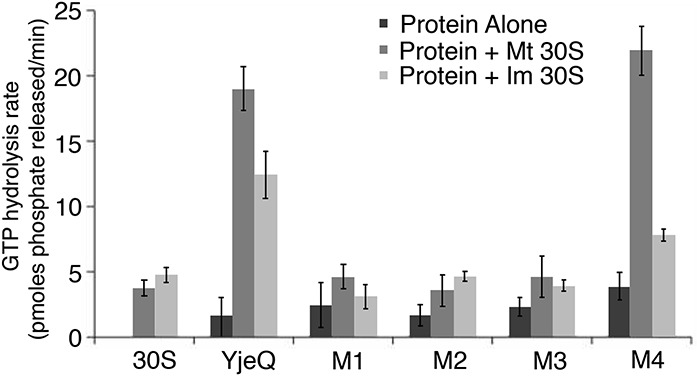
Stimulation of GTPase activity of YjeQ variants by mature and immature 30S subunits. The GTP hydrolysis rate of the YjeQ variants alone or in the presence of mature (Mt 30S) and immature 30S (Im 30S) subunits was assessed using the Malachite Green Phosphate assay as described in Materials and Methods. The GTPase hydrolysis rates plotted in the graph were determined by measuring the free phosphate produced after the reactions had been incubated for 60 min at 25°C. Standard deviations shown in the plot correspond to three replicas of the experiment.

We found that all the carboxy-terminal truncation variants of YjeQ exhibited an intrinsic initial rate of GTP hydrolysis comparable to full-length YjeQ and that ranged from 1–4 pmol/min (Fig. 2). When the mature 30S subunit was included in the reaction, the initial rate of the full-length YjeQ increased by approximately eightfold (see Materials and Methods). However, it did not increase considerably for any of the carboxy-terminal variants of YjeQ, except for the YjeQ M4 variant that showed an initial rate comparable to wild-type YjeQ. Using the immature 30S subunit in the reactions caused less than a threefold increase in the initial rate of full-length YjeQ, but again no increase was observed for the YjeQ variants (Fig. 2). This result is consistent with previous work (Himeno et al. 2004) that show the stimulation of the YjeQ GTPase activity by the immature 30S subunits is minimal in comparison to the mature 30S subunit. However, the fold-increase in the initial rate measured for the full-length protein was substantially lower than the 160-fold stimulation that had been reported (Daigle and Brown 2004). This difference is likely caused by the lower temperature (25°C versus 30°C) incubations in our assays.

Considering these results, we interpreted that the YjeQ M1 and YjeQ M2 variants did not show an increased level in 30S subunit-dependent GTPase activity (Fig. 2), possibly because both proteins cannot efficiently bind the mature 30S particle (Fig. 1B). In addition, we found that YjeQ M3 was able to bind the mature 30S subunit, but showed no increase in 30S subunit-dependent GTPase activity (Fig. 2). These results indicate that while the carboxy-terminal α-helix in the zinc-finger domain is not required for binding, it is a necessary motif for stimulation of GTP hydrolysis by the 30S subunit.

### YjeQ promotes the release of RbfA from the mature 30S subunit through the carboxy-terminal α-helix of the zinc-finger domain

Genetic and biochemical studies suggest that a function of YjeQ during maturation of the 30S subunit is assisting on the release of RbfA from the mature 30S subunit once the maturation of the particle is completed (Goto et al. 2011). It is not understood how this functional interplay is implemented and the structural determinants in YjeQ that allow the protein to perform this action. Prompted by the interesting phenotype of the YjeQ M3 variant that binds the 30S subunit without having an increase in its GTPase activity, we tested the ability of this YjeQ variant to remove RbfA and determined whether the CTE has any role in this activity.

To test the YjeQ M3 variant, we first modified the filtration assay to visualize the displacement of RbfA by full-length YjeQ. In these experiments, RbfA was first bound to the ribosomal subunits by incubating the reaction for 15 min. YjeQ was then added and incubated for an additional 15 min before the reaction was spun in the centrifugal device to separate free protein from that bound to the 30S subunits. The content in the flow through and bound portion was then analyzed by SDS-PAGE.

As in the assays testing the binding of YjeQ (Fig. 1B), the concentration of NH_4_Cl in the buffer is a critical parameter influencing the binding of RbfA to the small ribosome subunits. Performing the assembly reactions in buffers containing a range of NH_4_Cl concentration from 60 to 600 mM, we determined that at 60 mM NH_4_Cl, RbfA bound stoichiometrically to the immature 30S subunit (Supplemental Fig. S3, right panel). Binding of RbfA to immature 30S subunits is better than to mature 30S subunits (Goto et al. 2011), therefore this analysis was done with immature particles. In the case of mature 30S subunits, binding of RbfA is substoichiometric in buffer containing 60 mM NH_4_Cl (Supplemental Fig. S4A, middle panel). Consequently, the NH_4_Cl concentration in these assays aiming to visualize the release of RbfA upon binding of YjeQ was maintained at 60 mM NH_4_Cl, ensuring that binding of RbfA to the mature 30S subunit was still occurring (Supplemental Fig. S4, middle panel). We found that under these conditions YjeQ was able to efficiently remove RbfA from the mature 30S subunit. The removal of RbfA was very efficient in the presence of GTP (∼95%) and GMP-PNP (∼90%). In the presence of GDP, YjeQ displaced ∼65% of the RbfA bound to the mature 30S subunit (Supplemental Fig. S4A, middle panel).

In the case of immature 30S subunits, YjeQ could not release RbfA from the immature particle, regardless of the nucleotide present in the buffer (Supplemental Fig. S4A, bottom panel). To establish that the inability of YjeQ to release RbfA from the immature 30S subunit was not due to the low salt concentration on the buffer, this experiment was repeated also at higher salt concentration (Supplemental Fig. S4B). In this case the assembly reaction was carried out in the presence of GMP-PNP. We found that YjeQ still did not displace RbfA from the immature 30S subunits, even at the high salt concentrations and in the presence of GMP-PNP. Control filtration assays performed in parallel using YjeQ, RbfA, or both proteins in low or high salt buffer, showed that the filter did not retain the proteins by themselves or when combined in either buffer (Supplemental Fig. S4A, top panel; Supplemental Fig. S4B, left gel).

**FIGURE 3.**
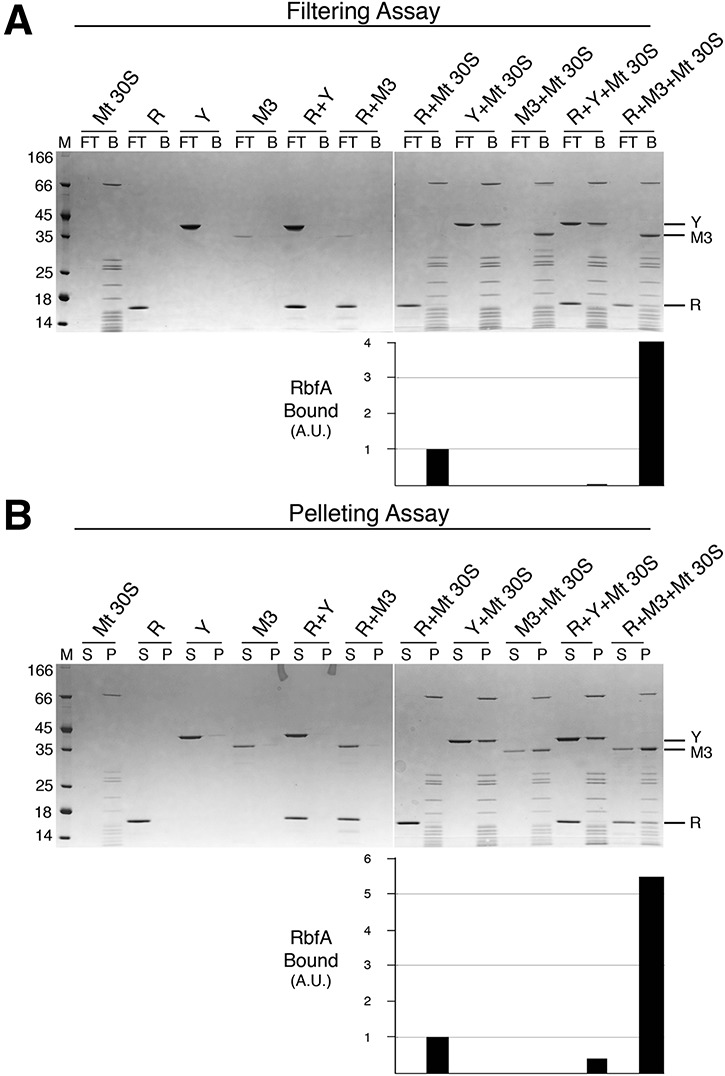
YjeQ M3 is unable to remove RbfA bound to the mature 30S subunit. (*A*) Filtration assay to test the release of RbfA (R) from the mature 30S subunit (Mt 30S) in the presence of YjeQ wild-type (Y) and YjeQ M3 variant (M3). These reactions were performed by adding the assembly factors in fivefold molar excess with respect to the 30S subunits and in the presence of GMP-PNP. Incubations were done at 25°C. Flow-through (FT) and bound (B) fractions from the filtration assay were resolved in 4%–12% bis–tris SDS-PAGE and stained with Coomassie blue. (*B*) Pelleting assay of identical reactions used in *A*. The molecular weight marker (M) is in kDa. Supernatant (S) and pellet (P) fractions from this assay were resolved in 4%–12% bis–tris SDS-PAGE and stained with Coomassie blue. The bar diagrams *under* the gels indicate the binding of the RbfA to the mature 30S subunit in each reaction. The observed binding of RbfA to the mature 30S subunit was defined as 1.

**FIGURE 4.**
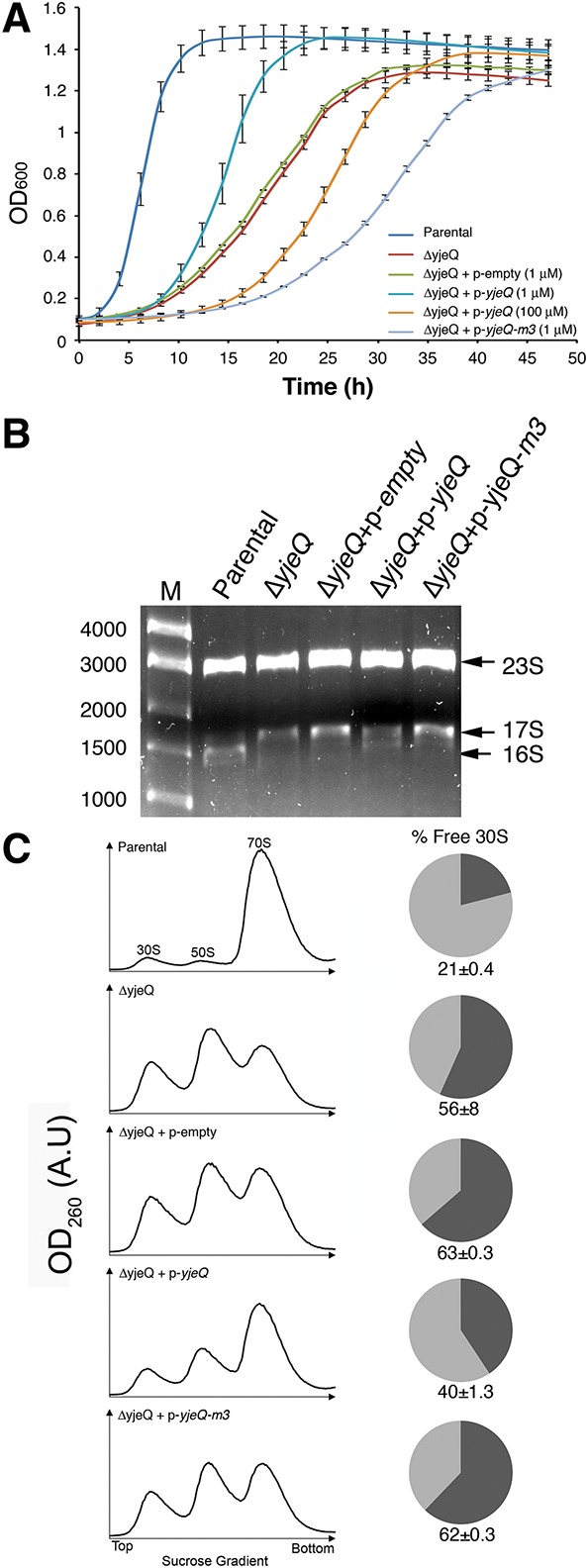
The carboxy-terminal extension of the zinc-finger domain in YjeQ is necessary for its function in vivo. (*A*) The parental strain and the *yjeQ* null strain by itself or complemented with the plasmid expressing yjeQ, YjeQ M3 variant, or the empty vector were induced at *t* = 0 h with the indicated concentration of IPTG and grown at 25°C for 47 h. Growth was monitored by measuring absorbance at 600 nm and plotted against time. Standard deviations shown in the plot correspond to three replicas of the experiment. (*B*) Total rRNA of cell cultures were analyzed when cultures reached mid-log phase represented by OD_600_ = 0.2. Total rRNA was extracted and resolved by electrophoresis in 0.9% synergel–0.7% agarose gel. The marker (M) is in base pairs. (*C*) Ribosome absorbance profiles from the parental strain and *yjeQ* null strain by itself or complemented with the plasmid expressing YjeQ, YjeQ M3 variant, or the empty plasmid were fractionated by ultracentrifugation in 10%–30% sucrose gradients providing the profiles shown in this panel. Peaks for the 30S, 50S subunits, and 70S ribosomes are indicated. The proportion of free 30S to bound 30S (i.e., 30S subunits in 70S complexes) in each case was calculated by integrating the areas *under* the 30S and 70S peaks of the sucrose gradient profiles. The area of the 30S peak plus one-third the area of the 70S peak corresponds to the total 30S population. The area of the 30S peak was divided by the total 30S absorbance to obtain the percentage of free 30S subunits and produce the pie charts in this panel. The standard deviations shown correspond to three replicas of the experiment. Peak area for the 30S subunit in each case was measured with respect to the area *under* the 70S peak to calculate the percentage of free 30S subunits in both strains. The calculated percentages are shown in the pie charts. The standard deviations shown correspond to three replicas of the experiment.

Now that a reliable assay was in place to visualize displacement of RbfA from the 30S subunit upon YjeQ binding, we tested the ability of the YjeQ M3 variant to perform this action. In this case, reactions were incubated at 25°C in order to maintain the solubility and folding of the YjeQ variant. Subsequently, the reaction was spun in a centrifugal device to perform the filtration assay. Interestingly, we found that whereas wild-type YjeQ removed 100% of the RbfA bound to the mature 30S subunit, the YjeQ M3 variant enhanced binding of RbfA by fourfold instead of removing it (Fig. 3A). Consistently with this result, when the reactions were laid over a sucrose cushion and subjected to a pelleting assay we observed a similar result (Fig. 4B). Wild-type YjeQ removed ∼60% of the RbfA bound to the mature 30S subunit and the YjeQ M3 variant increased binding of RbfA by 5.5-fold. These results indicate that the carboxy-terminal α-helix in the zinc-finger domain is the structural motif implementing the ability of YjeQ to remove RbfA from the 30S subunit once the maturation is completed. Furthermore, binding of the YjeQ M3 variant lacking the carboxy-terminal α-helix seemed to induce a conformation in the mature 30S subunit that stabilized the binding of RbfA.

### The carboxy-terminal extension of the zinc-finger domain in YjeQ is necessary for its function in vivo

The in vitro experiments described above indicated that the carboxy-terminal α-helix in the zinc-finger domain is an essential element for the 30S subunit-dependent GTPase activity of YjeQ (Fig. 2), as well as for YjeQ to promote on the release of RbfA once the maturation of the 30S subunit is completed (Fig. 3). To test whether the carboxy-terminal α-helix is necessary for YjeQ to assist in the assembly of the 30S subunits in vivo, we performed a complementation assay by expressing the YjeQ M3 variant in the *yjeQ* null strain. These cells were then tested for growth, rRNA and ribosome content. The wild-type *yjeQ* or *yjeQ m3* genes in these experiments were reintroduced in the null strain with a high-copy plasmid in which the expression is under the control of an IPTG-inducible T5 promotor (Kitagawa et al. 2005). Reintroduction of the gene in a plasmid, instead of in the chromosome eliminated the possibility of polar effects on downstream genes.

To analyze the growth, cells were grown at 25°C for up to 47 h and their optical density was measured and plotted over time (Fig. 4A). We also calculated their growth rate (Table 1). As previously described (Campbell et al. 2006; Goto et al. 2011) the *E. coli yjeQ* null strain exhibited a slow-growth phenotype (growth rate [GR] = 0.14 h^−1^) compared to the parental strain (GR = 0.44 h^−1^). We found that expression of wild-type YjeQ in small amounts achieved by using a low concentration (1 μM) of IPTG was sufficient to partially correct the slow-growth phenotype of the null *yjeQ* strain (GR = 0.21 h^−1^). However, overexpression of YjeQ by using a higher concentration of IPTG (100 μM) led the cells to grow slower than the null strain (GR = 0.12 h^−1^). This was likely the result of the toxic effect caused by an excess of YjeQ in the cells, which causes 70S subunits to dissociate (Daigle and Brown 2004; Himeno et al. 2004). Consequently to prevent the toxicity effects due to high levels of expression of YjeQ, the complementation experiments with the YjeQ M3 variant were performed using low concentrations of IPTG. Expression of the YjeQ M3 variant in this manner caused the cells to exhibit a growth rate of 0.10 h^−1^, which is even slower than that shown by the null *yjeQ* strain. In the control experiments where cells were transformed with the empty vector we found that it had no effect on the growth rate of the null strain (Fig. 4A; Table 1).

**TABLE 1.**
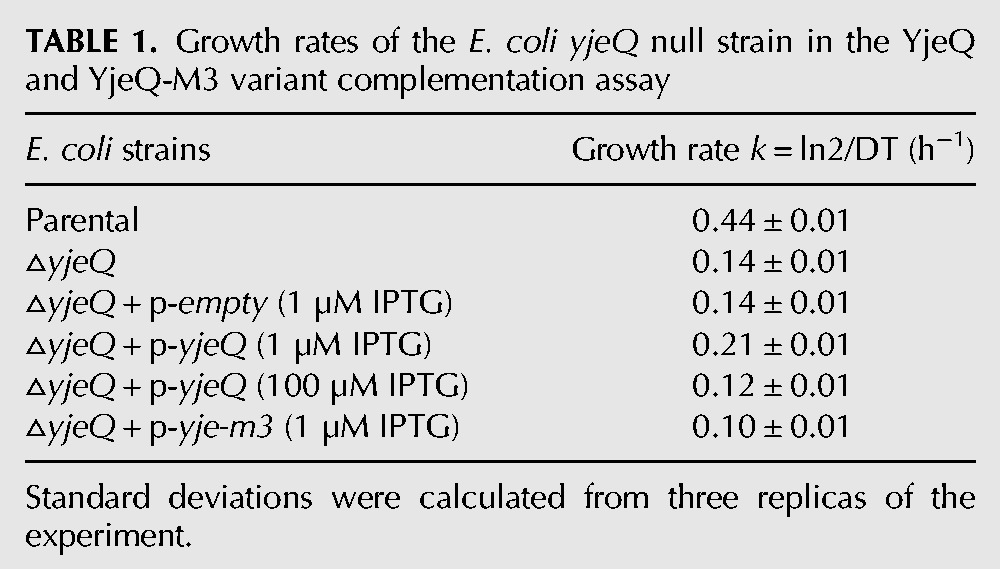
Growth rates of the *E. coli yjeQ* null strain in the YjeQ and YjeQ-M3 variant complementation assay

Total rRNA from the cells tested for growth was purified and resolved by gel electrophoresis (Fig. 4B) after they had been grown to mid-log phase. We found that the majority of the rRNA purified from parental cells was mature 16S rRNA, as opposed to the null *yjeQ* cells (alone or transformed with the empty plasmid), which mainly contained precursor 17S rRNA. Cells complemented with the plasmid expressing wild-type YjeQ showed that ∼50% of the accumulated rRNA had been processed into 16S rRNA. Conversely, in the cells expressing the YjeQ M3 variant most of the rRNA remained as unprocessed immature 17S rRNA (Fig. 4B).

Finally, we analyzed the ribosome profiles of the parental, null *yjeQ* cells and cells complemented with either the empty plasmid or that encoding wild-type YjeQ or the YjeQ M3 variant (Fig. 4C). The parental strain produced the expected profile with most of the particles assembled as 70S ribosomes and two small peaks representing dissociated 30S and 50S subunits. In these cells only ∼21% of the total 30S subunits were free and not associated with the 50S subunit. Null *yjeQ* cells and cells transformed with the empty plasmid produced a profile in which 56% and 63%, respectively, of the total 30S subunits did not associate to form 70S ribosomes (Daigle and Brown 2004; Himeno et al. 2004). Complementation with the plasmid expressing YjeQ caused the proportion of free 30S subunits to decrease to 40%. However, the percentage of free 30S subunits in cells expressing the YjeQ M3 was even higher (62%) than that of the null *yjeQ* cells. Therefore, we concluded from the analysis of the growth, rRNA, and ribosomal content that in vivo the YjeQ M3 variant was not able to complement the slow-growth phenotype exhibited by null *yjeQ* cells. Indeed, the observed phenotypes suggest that YjeQ M3 exhibited a gain-of function that is toxic most likely due to its enhanced binding to the 30S subunit and its ability to promote binding of RbfA (instead of facilitating release). Tighter binding of YjeQ and RbfA combined most likely impaired proper association of the 30S subunits with the 50S subunits to form 70S ribosomes. Consistently, we observed that the proportion of the free 30S subunits in the null *yjeQ* cells transformed with the plasmid expressing the YjeQ M3 was higher than that of the nontransfected null *yjeQ* cells.

## DISCUSSION

In this study, we found that the carboxy-terminal zinc-finger domain in YjeQ is important for the overall structural stability of the protein. Interestingly, an in silico analysis of other circularly permutated GTPases (Anand et al. 2006) concluded that a consequence of repositioning the G3/switch II motif within the GTPase domain is that an anchoring carboxy-terminal domain is then required to fasten switch II and maintain its efficiency for GTP binding and hydrolysis. Our finding that the carboxy-terminal zinc-finger domain is necessary for the stability of YjeQ is consistent with this model.

In addition, we also determined that the zinc-finger domain of YjeQ is essential for the role of this protein in assisting the late stages of maturation of the 30S subunit. The presented data indicate that this domain is subdivided into two functional motifs. The part of the domain coordinating the zinc ion is the region that allows for efficient binding of the protein to the 30S subunit, whereas the carboxy-terminal α-helix is the region through which YjeQ senses the binding to the mature 30S subunit triggering GTP hydrolysis in the GTPase domain. The zinc-finger domain is attached to the GTPase domain through the switch II region, which propagates the conformational change. In addition, the carboxy-terminal α-helix is also the region that implements the functional interplay of YjeQ with RbfA and facilitates the removal of RbfA from the mature 30S subunit once maturation of the 30S subunit is completed (Goto et al. 2011).

Recent structural studies with immature 30S subunits purified at late stages of assembly (Guo et al. 2011, 2013; Jomaa et al. 2011b; Boehringer et al. 2012; Clatterbuck Soper et al. 2013; Leong et al. 2013; Yang et al. 2014) revealed that the decoding center is the last region to fold into the mature conformation during the assembly of the subunit. Multiple assembly factors, including YjeQ, RbfA, RimM, and Era, bind to this region (Sharma et al. 2005; Datta et al. 2007; Guo et al. 2011; Jomaa et al. 2011b) at the late stages of assembly and operate in conjunction to monitor the proper folding of this region and prevent immature 30S subunits from engaging in translation. During this process functional interplays between assembly factors likely occur. In principle, any of the maturation steps these proteins facilitate including rRNA folding, rRNA processing, or mediating protein–RNA interactions could be assisted by several of these proteins simultaneously, rather than by protein factors individually. In addition, functional interplays likely play an important role in ensuring that all of the assembly factors are released once the maturation process is completed. Factor release is essential for the mature 30S subunit to enter the pool of actively translating ribosomes (Shajani et al. 2011).

Although there is genetic, biochemical, and structural evidence suggesting a functional interplay between these four putative assembly factors, the specific mechanism on how they work together to assist in maturation of the decoding center remains to be described. Recently, a study (Goto et al. 2011) identified that one of the functions of YjeQ is to assist in the release of RbfA once the maturation of the 30S subunit has been completed. However, it is still not understood how this functional interplay between the two proteins is implemented. A costructure of the 30S subunit in complex with both YjeQ and RbfA that could be informative about this mechanism has not yet been obtained. Furthermore, the cryo-EM structures of the YjeQ protein in complex with the mature 30S subunit (Guo et al. 2011; Jomaa et al. 2011b) propose two conflicting binding orientations of YjeQ to the 30S subunit. Therefore, an unambiguous functional model from these structural data cannot be derived.

**FIGURE 5.**
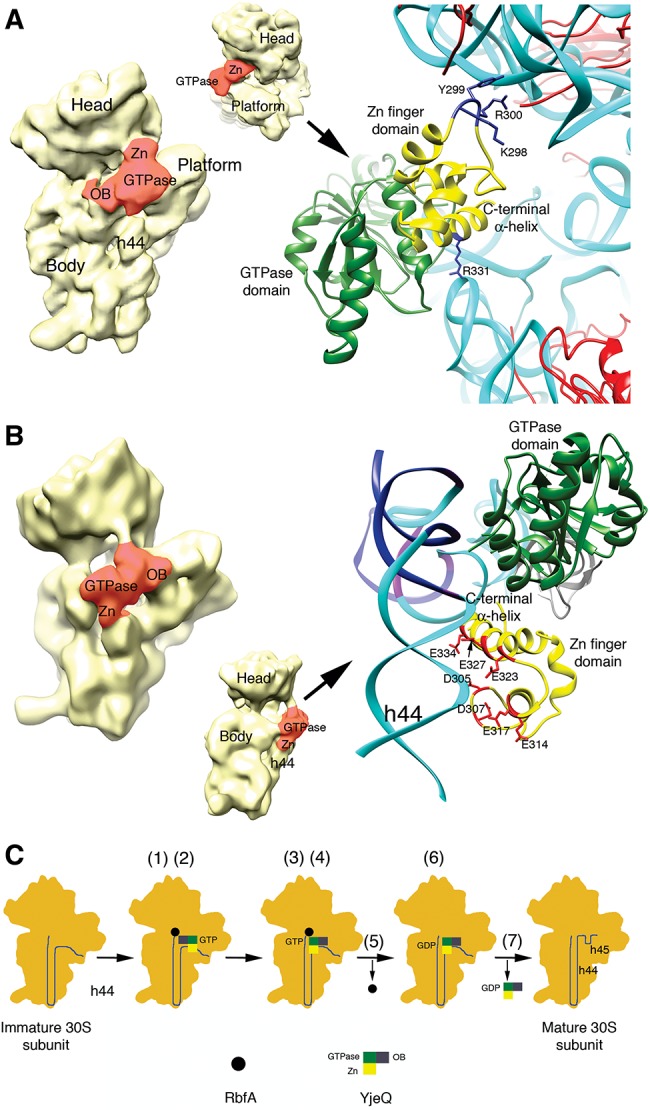
Interaction of the zinc-finger domain of YjeQ with the 30S subunit and working model for the functional interplay between YjeQ and RbfA. Structures of the 30S+YjeQ complex as described in Guo et al. (2011) (*A*) and Jomaa et al. (2011b) (*B*). The panel in the *left* shows a view of the entire complex. Landmarks and domains of the YjeQ protein are labeled. Area colored in red represents the YjeQ protein bound to the 30S subunit. The panel in the *right* shows a zoomed in view of the complex in the area of interaction of the zinc-finger domain with the 30S subunit. The rRNA is colored in cyan except for some nucleotides (*B*) that are colored in dark blue. A corresponding density for these nucleotides does not exist in the cryo-EM map of the 30S+YjeQ complex (Jomaa et al. 2011b). The side chains of important residues for this interaction are labeled. Structures shown were obtained from the EMDB (EMD-1884 and EMD-1895) and PDB (2YKR and 4A2I). Images of the structures were prepared with UCSF Chimera software (Pettersen et al. 2004). (*C*) Proposed model for the functional interplay between YjeQ and RbfA. The model explains the mechanism through which YjeQ facilitates the release of RbfA once the maturation of the 30S subunit is completed. Numbers in brackets refer to the steps of the model described in the text.

In this study, we found that the part of the domain coordinating the zinc ion in YjeQ is important for binding to the 30S subunit and the carboxy-terminal α-helix is essential for this factor to facilitate the release of RbfA from the mature 30S subunit. An important question is how these findings conform to the two conflicting cryo-EM structures (Guo et al. 2011; Jomaa et al. 2011b). The two cryo-EM structures showed that YjeQ binds to the 30S subunit covering the region of helix 44 forming the decoding center and also contacts the head and nearby region of the platform of the ribosomal particle (Fig. 5A,B). However, the two structures proposed a different orientation of the YjeQ protein in this complex. One of the structures (Guo et al. 2011) placed YjeQ with the OB-fold and GTPase domain contacting helix 44 and the carboxy-terminal zinc-finger domain interacting with the head (Fig. 5A, left panel). The second structure (Jomaa et al. 2011b) places YjeQ in an orientation rotated by ∼180° around an axis perpendicular to the interface surface of the 30S subunit. In this structure, the OB-fold domain is the region interacting with the platform and the carboxy-terminal zinc-finger domain sits in helix 44 (Fig. 5B, left panel).

Analysis of the two structures revealed that the data presented here are difficult to reconcile with the cryo-EM map where the zinc-finger domain contacts the head domain (Guo et al. 2011). According to this model, only three amino acids within the loop coordinating the zinc ion are close enough to interact with the phosphate–oxygen backbone of the rRNA in the head of the 30S subunit (Lys 298 and Arg 300) or r-protein S13 (Tyr 299) (Fig. 5A, right panel). In this structure, the rest of the zinc-finger domain, including the carboxy-terminal α-helix does not directly contact with either the head or the platform. Only Arg 331 in the carboxy-terminal α-helix comes close to the backbone of helix 24 (Fig. 5A, right panel). Our mutational study indicates that the part of the domain coordinating the zinc ion is essential for YjeQ to bind the mature 30S subunit. Additionally, we found that mutating two (Lys 298 and Arg 300) out of the three residues that come into close contact with the head of the 30S subunit did not abolish binding (Fig. 1B). Therefore, it is difficult to envision how the carboxy-terminal α-helix could facilitate the release of RbfA from the mature 30S subunit with the zinc-finger domain in this location and having minimal interactions with the ribosome (Fig. 5A, right panel).

In the second cryo-EM structure (Jomaa et al. 2011b), the zinc-finger domain of YjeQ sits in the upper domain of helix 44 near the decoding center (Fig. 5B). In this location, negatively charged residues in the surface of the loop region coordinating the zinc ion and more importantly the carboxy-terminal α-helix face the negatively charged phosphate–oxygen backbone of nucleotides in helix 44 (Fig. 5B, right panel). This charge distribution in the two motifs of the zinc-finger domain generates electrostatic repulsion with helix 44 causing a displacement of the upper domain of the helix from its mature conformation (Fig. 5B, right panel). It is possible that the carboxy-terminal α-helix in YjeQ facilitates the release of RbfA from the mature 30S subunit by preventing the upper domain of helix 44 to adopt the mature conformation.

RbfA binds in the neck region of the 30S subunit (Datta et al. 2007) also causing a displacement of the upper domain of helix 44 and helix 45. The cryo-EM structure of the 30S subunit in complex with RbfA shows that upon binding, these motifs surround RbfA and lock the protein deeply into the cleft between the head and body. Considering that complexes in this cryo-EM study were prepared with a 10- to 40-fold excess of RbfA with respect to the 30S subunits (Datta et al. 2007), it is plausible that the conformation visualized by cryo-EM represent a “trapped” state of RbfA bound to the mature 30S subunit with the displaced helix 44 and 45 blocking the release of the factor. One of our findings in this study was that substantial binding of YjeQ to the immature 30S subunit is still observed in the absence of the zinc-finger domain (Fig. 1C). Taking all these data into consideration, we propose the following model (Fig. 5C) to explain the functional interplay between YjeQ and RbfA in mediating the maturation of the decoding center. (1) RbfA first binds in the neck region of the immature 30S subunit, which at this point still exhibits the part of helix 44 forming the decoding center in an immature state. (2) RbfA assists in the recruitment of YjeQ, which initially binds to the 30S subunit largely through the OB-fold domain. (3) As the upper domain of helix 44 continues to fold into its mature state, the region in this helix forming the binding site for the zinc-finger domain of YjeQ adopt the proper conformation to allow YjeQ to bind the 30S subunit through both the OB-fold and zinc-finger domains. This is likely the conformation shown by one of the cryo-EM structures (Jomaa et al. 2011b). (4) Binding of YjeQ through the zinc-finger domain to the 30S subunit prevents the upper domain of helix 44 to fold over the bound RbfA and lock this factor in the “trapped” state shown by the cryo-EM map of the 30S+RbfA complex (Datta et al. 2007). (5) By maintaining the upper domain of helix 44 in a non-native conformation, YjeQ facilitates the release of RbfA. (6) Release of RbfA is sensed by the carboxy-terminal α-helix, activating hydrolysis of the GTP molecule bound to YjeQ. (7) The reduced affinity of YjeQ-GDP for the 30S facilitates the dissociation of YjeQ from the mature subunit through a still uncharacterized mechanism, allowing the upper domain of helix 44 to adopt the conformation observed in the decoding center of the mature subunit. Dissociation of YjeQ also uncovers essential intersubunit bridges (B2a and B3) allowing the mature 30S subunit to associate with the 50S subunit and engage in protein translation.

An important consideration regarding our experimental set up to analyze the YjeQ-induced release of RbfA is that it does not completely mimic the sequence of events most likely occurring in the cell (Fig. 5C). Mainly, in our assays we bound RbfA directly to the mature 30S subunit and then YjeQ was added to test RbfA release. In the cell, it is most likely that RbfA would bind to the immature 30S subunit (instead to the mature 30S subunit) and YjeQ-induced release would take place once this subunit has evolved and becomes a mature subunit. Unfortunately, attempts of starting our reactions with the immature 30S subunit in complex with RbfA and maturing these subunits in vitro to then test the release of RbfA with YjeQ were unsuccessful. Nevertheless, it is likely that the binding mode of RbfA to the mature 30S subunit in our in vitro assays closely resembles that existing in the analogous complex in vivo. Consequently, conclusions withdrawn from these binding assays most likely apply to the maturation process occurring in vivo. In this regard, future efforts to determine the structure of mature and immature 30S subunits in complex with RbfA and YjeQ will be important to visualize the conformational changes suggested by the model described above and how the implementation of the functional interplay between YjeQ and RbfA occurs.

## MATERIALS AND METHODS

### Cell strains and protein overexpression clones

Parental *Escherichia coli* K-12 (BW25113) and *E. coli* Δ*yjeQ* strains were obtained from the Keio collection (Baba et al. 2006).

The pDEST17-*yjeQ* plasmid used to overexpress wild-type YjeQ protein with an amino-terminal His_6_ tag cleavable by TEV protease was produced as previously described (Jomaa et al. 2011b). The pDEST17-*yjeQM1*, pDEST17-*yjeQM2*, pDEST17-*yjeQM3*, and pDEST17-*yjeQM4* plasmids used to overexpress the YjeQ carboxy-terminal deletion variants were generated by using the QuikChange II XL Site-Directed Mutagenesis Kit (Agilent Technologies). To this end, the parental pDEST17-*yjeQ* plasmid was used as the template for site-directed mutagenesis reactions that introduced a stop codon in the position of L278 (YjeQM1), F287 (YjeQM2), K320 (YjeQM3) or to create the point mutations K298A and R300A (YjeQM4). The Q5 Site-Directed Mutagenesis Kit (New England Biolabs) was used to produce the pDEST17-*yjeQM5* plasmid containing mutations C297A and C310A. In this case, we used the same template plasmid as the other mutants.

Overexpression of RbfA was obtained using the pET15b-*rbfA* plasmid. To produce this plasmid the sequence of the *rbfA* gene (NCBI reference: NC_007779.1) was optimized for overexpression in *E. coli* cells using the GeneOptimizer software and subsequently synthesized (Life Technologies; Thermo Fisher Scientific) with a NdeI and a BamHI site in the 5′ and 3′ ends of the gene, respectively. The gene was cloned into the carrier pMA-T plasmid using the SfiI and SfiI cloning sites and subsequently subcloned into the final expression vector pET15b using the NdeI and a BamHI restriction sites. The resulting pET15b-*rbfA* plasmid produces the RbfA protein with an amino-terminal His_6_ tag cleavable by thrombin.

We used sequencing (MOBIX, McMaster University) to validate all overexpression clones.

### Protein overexpression and purification

Wild-type YjeQ protein and the constructed variants (YjeQ M1, YjeQ M2, YjeQ M3, YjeQ M4, and YjeQ M5) were overexpressed as amino-terminal His_6_-tag proteins by transforming *E. coli* BL21-A1 cells with the corresponding expression plasmids constructed to produce these proteins (see above). Typically, 1 L of cells were grown in LB medium at 37°C to an OD_600_ of 0.6 and expression was induced with 0.2% L-arabinose. Wild-type YjeQ and YjeQ M4 were induced for 3 h at 37°C, whereas YjeQ M1, YjeQ M2, and YjeQ M3 were induced for 16 h at 16°C due to the instability and low solubility of the proteins at 37°C. In the case of the YjeQ M5 variant, expression was tested at 16 h at 16°C, 5 h at 25°C, and 3 h at 37°C. Cells were harvested after induction by centrifugation at 8500*g* for 15 min. The cell pellets were then washed with 1× PBS buffer (137 mM NaCl, 2.7 mM KCl, 8.1 mM Na_2_HPO_4_, 1.76 mM KH_2_PO_4_ at pH 7.4), centrifuged at 1400*g* for 20 min and resuspended in 20 mL of lysis buffer (50 mM Tris–HCl at pH 8.0, 10% [w/v] sucrose, 100 mM NaCl). The cell suspension was passed through the French press three consecutive times at 20,000 lb/in^2^ to lyse the cells. Lysate was separated from cell debris by centrifugation at 30,000*g* for 40 min and NaCl was added to the clarified lysate to bring the concentration to 0.5 M. The lysate was then filtered with a 0.45 µm filter and loaded onto a HiTrap Metal Chelating column (GE Healthcare Life Sciences) equilibrated with 50 mM Tris–HCl at pH 8.0, 0.5 M NaCl and 5% [v/v] glycerol. The column was washed with buffer containing concentrations of imidazole of 45 mM and 90 mM. Finally, proteins were eluted by increasing the concentration of imidazole in the buffer to 240 mM. The purity of fractions was monitored by 15% SDS-PAGE. Fractions with pure protein were collected and dialyzed overnight at 4°C against buffer containing 50 mM Tris–HCl at pH 8.0 and 5% [v/v] glycerol. For YjeQ M1, YjeQ M2, and YjeQ M3, the dialysis buffer also included 120 mM NaCl to increase protein solubility.

The amino-terminal His_6_-tag was removed by digestion with purified tobacco etch virus (TEV) protease during the previous overnight dialysis step. To this end, 0.05 mg of TEV protease per milligram of YjeQ was added to the pooled and dialyzed fractions containing the wild-type or YjeQ variants. Following digestion and dialysis, the reaction was loaded onto the HiTrap Metal Chelating column (GE Healthcare Life Sciences) previously equilibrated with 50 mM Tris–HCl at pH 8.0, 60 mM imidazole, 0.2 M NaCl, and 5% [v/v] glycerol. The flow through containing the untagged protein was recovered and purity of fractions showing cleaved protein was monitored by 15% SDS-PAGE. Fractions with pure, cleaved protein were dialyzed overnight at 4°C against buffer containing 50 mM Tris–HCl at pH 8.0 and 5% [v/v] glycerol. For YjeQ M1, YjeQ M2, and YjeQ M3 the dialysis buffer also included 120 mM NaCl. Pure proteins were concentrated using a 10 kDa-cutoff filter (Amicon). Proteins were frozen in liquid nitrogen and stored at −80°C.

The RbfA protein containing an amino-terminal His_6_-tag was overexpressed in *E. coli* BL21-DE3 cells transformed with the pET15b-*rbfA* plasmid. One liter of cells was grown in LB medium at 37°C to an OD_600_ of 0.6 and then induced with 1 mM isopropyl β-D-1-thiogalactopyranoside (IPTG) for 3 h at 37°C. Following induction, cells were harvested and lysed using the same protocol and buffers as in the case of the YjeQ protein. The first step of purification of RbfA included a HiTrap Metal Chelating column (GE Healthcare Life Sciences) that was performed in an identical manner to the YjeQ purification except that the column washes before elution were done with buffers containing 30 and 75 mM imidazole. Fractions containing RbfA protein were collected, pooled, and dialyzed overnight at 4°C against buffer containing 50 mM Tris–HCl at pH 8.0 and 5% [v/v] glycerol. Dialyzed RbfA was then loaded onto a HiTrap Q HP Anion Exchange column (GE Healthcare Life Sciences) previously equilibrated with 50 mM Tris–HCl at pH 8.0 and 5% [v/v] glycerol. Nonspecifically bound proteins were washed with 50 mM NaCl and RbfA was eluted by increasing the NaCl concentration to 100 mM. Purity of the fractions was monitored by 15% SDS-PAGE. Fractions with pure RbfA were dialyzed overnight at 4°C against buffer containing 50 mM Tris–HCl at pH 8.0 and 5% [v/v] glycerol and concentrated using a 10 kDa-cutoff filter (Amicon). Purified RbfA was frozen in liquid nitrogen and stored at −80°C. The amino-terminal His_6_-tag in RbfA was not removed in order to facilitate visualization of the protein in the binding assays with the 30S ribosomal subunits (see below).

### Purification of 30S ribosomal subunits

Parental (BW25113) and Δ*yjeQ E. coli* cells were used as the source for purification of the mature and immature 30S ribosomal subunits. We used centrifugation over sucrose cushions and gradients as previously described (Jomaa et al. 2011a) to purify both ribosomal particles from these cells (Supplemental Fig. S5A). In the case of the mature 30S subunits, sucrose gradients loaded with ribosomal particles from parental cells were resolved under “dissociating” conditions (buffer containing 1.1 mM magnesium acetate) that leads to the dissociation of the 70S subunits and allows for an efficient purification of mature 30S subunits. Under “associating conditions” (buffer containing 10 mM magnesium acetate), the parental strain yielded a characteristic ribosome profile with the majority of 30S subunits assembled as mature 70S particles, establishing that these cells have a normal wild-type phenotype (Supplemental Fig. S5A). Immature 30S subunits were purified from Δ*yjeQ*. In this case, sucrose gradients loaded with purified ribosomes were resolved under “associating conditions.” These cells typically accumulate a large proportion of free immature 30S subunits (∼39%) (Supplemental Fig. S3A), thus these conditions allowed us to obtain a homogeneous preparation of immature subunits without contaminating mature 30S subunits.

The rRNA analysis of 30S subunits from parental and Δ*yjeQ E. coli* cells was performed to verify that our purifications of 30S subunits consisted of mature and immature 30S subunits, respectively (Supplemental Fig. S5B). For each preparation, we took ∼10–50 pmol of purified ribosome subunits and used the RNeasy Mini Kit (Qiagen) to purify the rRNA following manufacturer's protocols. Subsequently, purified rRNA samples from the fractions corresponding to the 30S, 50S, and 70S peaks in the sucrose gradients were resolved in a 0.9% synergel–0.7% agarose gel using previously described methods (Wachi et al. 1999). This approach allows for the separation during electrophoresis of the 23S, 17S, and 16S rRNAs during electrophoresis. The preparations of mature and immature 30S subunits did not contain any 23S rRNA indicating the absence of 50S subunit contamination. The purification of 30S subunits from parental cells showed the presence of 16S rRNA indicating that it mainly contained mature 30S subunits. Instead, the 30S subunits purified from Δ*yjeQ* cells contained exclusively 17S rRNA, which is the precursor form of the mature 16S rRNA, indicating that the 30S subunits in these preparations were immature. The Δ*yjeQ* cells also contained small amounts of 16S rRNA, but it was incorporated into 30S subunits that were associated to the 50S subunit and appeared in the 70S peak of the gradient.

### Circular dichroism spectroscopy

CD spectra for each YjeQ protein were collected using a Circular Dichroism Spectrometer Model 410 (Aviv Biomedical, Inc.). Spectral scans were performed from 260 to 200 nm, with step resolution and bandwidth of 1.0 nm. A 1-mm-path-length quartz cuvette was used for the measurements. The spectra for each protein and each temperature (4°C, 16°C, 25°C, and 37°C) are an average from three consecutive scans obtained during a 15-min incubation period. Protein solutions were prepared at a concentration of 0.5 mg/mL in Binding Buffer 300 (10 mM Tris–HCl at pH 8.0, 7 mM magnesium acetate, 300 mM NH_4_Cl, and 1 mM dithiothreitol [DTT]). The machine units of millidegrees ellipticity were used to calculate the mean residue molar ellipticity in [θ] using the following equation:

$$\begin{array}{rl} \left[\theta ](\deg\cdot {\mathrm{cm}}^{2}∙{\mathrm{dmol}}^{-1}\right)\\ & =\mathrm{Ellipticity}(\mathrm{mged})∙10^{6}/\mathrm{Pathlength}(\mathrm{mm})\\ & \;∙[\mathrm{Protein}](\mathrm{mM})∙n \end{array}$$

(*n* is the number of peptide bonds in the protein).

### Binding assays

Reactions for filtration assays to detect binding of YjeQ and RbfA to mature and immature 30S subunits were prepared by mixing 200 pmol of protein (YjeQ, YjeQ variants, and/or RbfA) with 40 pmol of 30S subunit in a 100 µL reaction in either Binding Buffer 60 (10 mM Tris–HCl at pH 8.0, 7 mM magnesium acetate, 60 mM NH_4_Cl, 1 mM DTT, and 1 mM GMP-PNP) or Binding Buffer 300 (10 mM Tris–HCl at pH 8.0, 7 mM magnesium acetate, 300 mM NH_4_Cl, 1 mM DTT, and 1 mM GMP-PNP). The concentration of each protein assembly factor in this reaction was fivefold that of the ribosomal particle. Nucleotide (GMP-PNP, GDP, or GTP) was added where indicated to a final concentration of 1 mM. Reactions were incubated at 16°C, 25°C, or 37°C for 15 min followed by centrifugation in a 100 kDa Nanosep Centrifugal Devices (PALL). In cases where YjeQ was added following a 15 min incubation of RbfA with the ribosomal particle, the binding reaction was allowed to proceed for another 15 min. Prior to loading the binding reactions in the 100 kDA Nanosep Centrifugal Devices (PALL), its filter membrane was blocked by loading 500 µL of 1% [w/v] BSA and washed twice with 500 µL of RNase free water. Reactions were spun at 12,000*g* for 10 min to separate 30S particles and 30S-bound proteins that were retained by the filter from unbound proteins in flow-through (FT). The flow-through was collected and the filter was washed gently twice with 100 µL of Binding Buffer 60 or 300, followed by a 5 min spin at 12,000*g*. Finally, the 30S particles and 30S-bound proteins retained by the filter were vigorously resuspended in 100 µL of Binding Buffer 60 or 300 and collected as the bound fraction (B). To resolve the flow-through and bound fractions, 30 µL of sample were mixed with 6× SDS-PAGE loading buffer and loaded into a 4%–12% Criterion XT Bis–tris gel (Bio-Rad). Samples were run in XT MOPS buffer (Bio-Rad). Gels were stained with Coomassie blue.

In the case of the pelleting assays, reactions were prepared by mixing 250 pmol of protein (YjeQ, YjeQ variants, and/or RbfA) with 50 pmol of 30S particle in a 50 µL reaction in either Binding Buffer 60 or Binding Buffer 300. The final concentration of GMP-PNP in the binding reactions containing YjeQ and YjeQ variants was 2 mM. Reactions were incubated at 16°C, 25°C, or 37°C for 15 min. In cases where RbfA was added following a 15 min incubation of YjeQ with the ribosomal particle, the binding reaction was allowed to proceed for another 15 min. Following incubation, reactions were laid over a 150 µL 1.1 M sucrose cushion in either Binding Buffer 60 or 300 and spun for 250,000*g* for 5 h. The supernatant (S) containing free protein that did not pellet with the 30S subunits was collected. The pellet (P) containing the 30S particles and 30S-bound proteins was resuspended in 200 µL of Binding Buffer 60 or 300. To resolve the supernatant and pellet fractions, 30 µL of sample was mixed with 6× SDS-PAGE loading buffer and loaded into a 4%–12% Criterion XT Bis–tris gel (Bio-Rad). Samples were run in XT MOPS buffer (Bio-Rad). Gels were stained with Coomassie blue.

Image Lab (V5) software (Bio-rad) was used to perform a densitometry analysis on the Coomassie stained gels for relative quantification of assembly factors binding to 30S subunits. Specifically, the lanes were automatically detected using a background subtraction with a disc size of 5 mm. Bands were then automatically detected using high sensitivity settings (sensitivity: 5, size scale: 7, noise filter: 4, shoulder: 1) followed by manual adjustment of both lanes and bands to ensure accurate measurements. The quantity tools implemented with the Image Lab software package were used to determine the relative amounts of each assembly factor in the bound portion (YjeQ or RbfA) to the S4 protein within the same lane. Binding of wild-type YjeQ or RbfA to the mature or immature 30S subunit was considered as 1 and all other binding interactions were quantified with respect to this value. Prior to the quantification of the bands for YjeQ, YjeQ, variants and RbfA, their intensity was normalized to account for small differences in the amount of binding reaction loaded in the gel. To this end, we used the band for r-protein S4, which should have constant intensity in all binding reactions regardless of whether mature or immature 30S subunits were used. The r-protein S4 is not partially depleted in the immature 30S subunits purified from the Δ*yjeQ E. coli* cells.

### GTPase activity assays

The intrinsic initial rates of GTP hydrolysis of the full-length YjeQ and YjeQ variants were measured by incubating each protein (100 nM) with GTP (250 µM) at 25°C for 60 min and then measuring the release of free phosphate using the Malachite Green Phosphate Assay Kit (BioAssay Systems). The reactions were initiated by adding GTP to the proteins in reaction buffer containing 10 mM Tris–HCl at pH 8.0, 7 mM magnesium acetate, 300 mM NH_4_Cl, and 1 mM DTT. In those cases where the stimulation of the GTPase activity of YjeQ or YjeQ variants by the 30S particles was measured, the sample also contained 100 nM of 30S particle before GTP was added. Volume of the reactions was 80 µL. Reactions were terminated by addition of 20 µL of the malachite green reagent and an additional incubation of 15 min at 25°C before monitoring color formation by measuring the absorbance at 620 nm. Values were plotted against a standard curve of free phosphate in the same reaction buffer. The amount of phosphate produced by the reaction containing only buffer and GTP was considered background and was subtracted from each reaction. To calculate the fold-increase of the initial rate, the background level of GTP hydrolysis of the 30S particle by itself was subtracted from the value obtained for the reaction containing YjeQ and the 30S subunit. Reactions were run in a 96-well plate and readings were done with the Infinite M1000 multiplate reader (TECAN). Average and standard deviation values were derived from three replicas of each reaction. We determined that under the conditions used to measure GTPase activity, the amount of free phosphate produced in the reactions was within the linear range of the assay.

### Culture growth conditions

The growth rate of the null *yjeQ* and parental *E. coli* (BW25113) strains was determined by growing the cells at 25°C for 47 h with shaking. Culture density was monitored by measuring the optical density at 600 nm (OD_600_) in a Sunrise 96-well plate reader (TECAN) and plotted using Magellan (TECAN). Doubling times were calculated according to the formula DT = (*t*2 − *t*1) × [log2/(log OD_600_@*t*2/log OD_600_@*t*1)] and expressed in hours. Growth rates were calculated as *k* = ln2/DT and expressed in h^−1^.

The growth curves were produced by inoculating 200 µL of LB media from overnight cultures at 1:100 dilutions. To reintroduce the YjeQ or YjeQ M3 variant, we used the high-copy plasmid pCA24N. The pCA24N-empty and pCA24N-*yjeQ* plasmids were purified from the ASKA collection, which contains a complete set of open reading frame clones of *E. coli* (Kitagawa et al. 2005). The pCA24N-*yjeQ* plasmid expresses the amino-terminal histidine-tagged YjeQ under the control of the IPTG-inducible promoter P_*T5-lac*_. The pCA24N-*yjeQM3* plasmid was constructed from the pCA24N-*yjeQ* plasmid using QuikChange II XL Site-Directed Mutagenesis Kit (Agilent Technologies) to introduce a stop codon in the position of Lys 320. We used standard protocols (Sambrook et al. 1989) to transform these plasmids into the Δ*yjeQ* cells before proceeding to obtain the growth curves as described above. In the cultures where we wanted to express YjeQ or the YjeQ M3 variant (or pCA24N-empty plasmid) the LB media contained either 1 µM or 100 µM IPTG as specified.

To obtain the ribosome profiles and analyze the rRNA content of these cells, LB cultures (1 L) were grown at 25°C to an OD_600_ of 0.2. In the case of the strains containing the pCA24N-empty plasmid or that encoding for YjeQ or YjeQ M3 variant, we added IPTG to a concentration of 1 µM. Following growth, 10 mL of culture were pelleted by centrifugation at 1400*g* for 20 min and processed to purify the rRNA. Total rRNA was analyzed by extracting rRNA from the cell pellet with the RNeasy Mini Kit (Qiagen). The concentration of purified rRNA was measured by absorbance at *A*_260_, where 1 absorbance unit was equivalent to 40 µg/mL of RNA. Approximately 0.8 µg of purified rRNA was loaded on a 0.9% synergel–0.7% agarose gel, using methods described previously to separate 23S, 17S, and 16S rRNA (Wachi et al. 1999).

The remainder of the cultures were harvested by spinning the cells at 8500*g* for 15 min to obtain ribosome profiles. Pellets were washed and resuspended with 20 mL of Buffer A (10 mM Tris–HCl at pH 7.5, 10 mM MgCl_2_, and 60 mM KCl), divided into three conical tubes and then centrifuged at 1400*g* for 15 min. Each pellet was resuspended in 6 mL of Lysis buffer (10 mM Tris–HCl at pH 7.5, 10 mM MgCl_2_, and 60 mM KCl, 0.5 % [v/v] Tween 20, 1 mM DTT, 1 tablet/10 mL complete mini Protease Inhibitor Cocktail [Roche]), and 20 µL of RNase Free DNAse (Invitrogren). The cell suspension was lysed by passing it through the French press three consecutive times at 20,000 lb/in^2^ and clarified by centrifuging at 19,000*g* for 10 min. Ribosomes were pelleted by centrifuging the clarified lysate at 125,000*g* for 1 h 52 min. The ribosomal pellet was rinsed with 1 mL of Buffer B (20 mM Tris–HCl at pH 7.5, 6 mM MgCl_2_, 30 mM NH_4_Cl, and 1 mM DTT) and resuspended in 3 mL of this same buffer for 30 min on ice. An equal amount of Buffer C (20 mM Tris–HCl at pH 7.5, 6 mM MgCl_2_, 800 mM NH_4_Cl, and 1 mM DTT) was added and further incubated on ice for 1 h. The mixture was clarified by centrifuging at 19,000*g* for 10 min and subsequently ribosomes were pelleted by centrifuging at 125,000*g* for 1 h 52 min. The ribosomal pellet was rinsed with 700 μL of Buffer D (20 mM Tris–HCl at pH 7.5, 10 mM MgCl_2_, 30 mM NH_4_Cl, and 1 mM DTT) and resuspended in 700 μL of the same buffer for 1 h. The mixture was clarified by centrifuging at 19,000*g* for 10 min and 10 A_260_ units were laid on top of 10 mL 10%–30% sucrose gradients made with Buffer E (20 mM Tris–HCl at pH 7.5, 10 mM MgCl_2_, 50 mM NH_4_Cl, and 1 mM DTT). Gradients were centrifuged at 43,000*g* for 16 h using a Beckman SW41 Ti swinging-bucket rotor. Gradients were then fractioned using a Brandel fractionator apparatus and an AKTAprime purification system (GE Healthcare). The elution peaks of the ribosomal subunits were monitored by absorbance at *A*_260_.

## SUPPLEMENTAL MATERIAL

Supplemental material is available for this article.

## Supplementary Material

Supplemental Material
