# Supplementary material for: The C-terminal helix in the YjeQ zinc-finger domain catalyzes the release of RbfA during 30S ribosome subunit assembly
Source: RNA. 2015 Jun;21(6):1203–16. doi: 10.1261/rna.049171.114 (PMC4436671; doi:10.1261/rna.049171.114)
Supplement: Supplemental Material [file supp_21_6_1203__index.html]

The C-terminal helix in the YjeQ zinc-finger domain catalyzes the release of RbfA during 30S ribosome subunit assembly — The C-terminal helix in the YjeQ zinc-finger domain catalyzes the release of RbfA during 30S ribosome subunit assembly — Supplemental Material 

# The C-terminal helix in the YjeQ zinc-finger domain catalyzes the release of RbfA during 30S ribosome subunit assembly

## Supplemental Material

**Files in this Data Supplement:**

- Supp Material.pdf
