# Supplementary material for: The C-terminal helix in the YjeQ zinc-finger domain catalyzes the release of RbfA during 30S ribosome subunit assembly
Source: RNA. 2015 Jun;21(6):1203–16. doi: 10.1261/rna.049171.114 (PMC4436671; doi:10.1261/rna.049171.114)
Supplement: Supplemental Material [file supp_049171.114_SuppMaterial.pdf]

The C-terminal helix in the YjeQ zinc finger domain catalyzes the release of RbfA during 30S ribosome subunit assembly

Ajitha Jeganathan, Aida Razi, Brett Thurlow and Joaquin Ortega*

Department of Biochemistry and Biomedical Sciences and M. G. DeGroote Institute for Infectious Diseases Research, McMaster University, 1280 Main Street West, Hamilton, Ontario, L8S4K1, Canada.

This supplement contains:

Supplemental Figures S1 to S5

SUPPLEMENTAL FIGURES

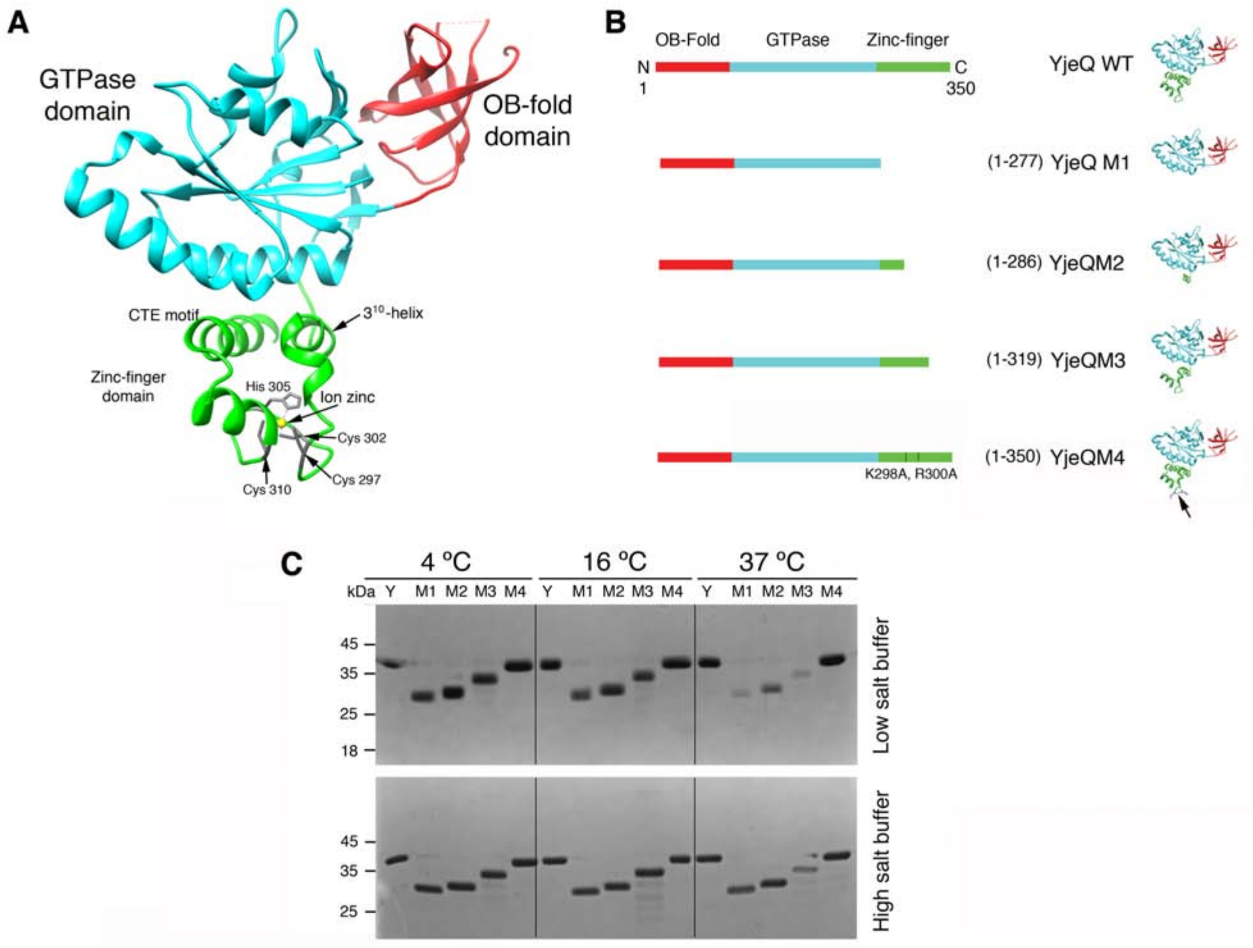

Figure S1. Stability of the C-terminal variants of YjeQ. (A) X-ray structure of YjeQ from *Salmonella typhimurium* (PDB ID 2RCN). The three domains of the protein and important landmarks of the zinc-finger domains are indicated. (B) (Left) Diagrams describing the primary structure of YjeQ and the constructed C-terminal variants of YjeQ. (Right) To easily visualize in 3D the part of the zinc finger domain removed in each YjeQ variant, the deleted amino acid sequence was not displayed in the X-ray structure of *S. typhimurium* YjeQ (PDB ID 2RCN). In the case of YjeQ M4 the arrow in the structure indicates the position of Lys 298 and Arg 300 that were both mutated to alanine. (C) Comparison of the stability of the YjeQ variants with that of the wild type using precipitation tests. Equal amounts of wild type YjeQ and YjeQ variants were

incubated for 15 minutes at 4 °C, 16 °C or 37 °C in buffer containing either low (60 mM) or high salt (300 mM NH_4Cl). Following incubation, samples were spun down at 12000*g* for 5 minutes and the non-precipitated fraction of the protein was estimated by resolving the supernatant in a 15% SDS-PAGE stained with Coomassie blue (Supplemental Fig. S1C). The amount of YjeQ variants recovered as soluble equated to that of wild type YjeQ after incubation at 4 °C and 16 °C in both low and high salt buffers. However, when the incubation was performed at 37 °C all three YjeQ variants appeared to be unstable in the buffer containing low salt (upper panel). Under high salt conditions, the YjeQ M1 and M3 variants also precipitated partially, but to a much lesser extent (lower panel).

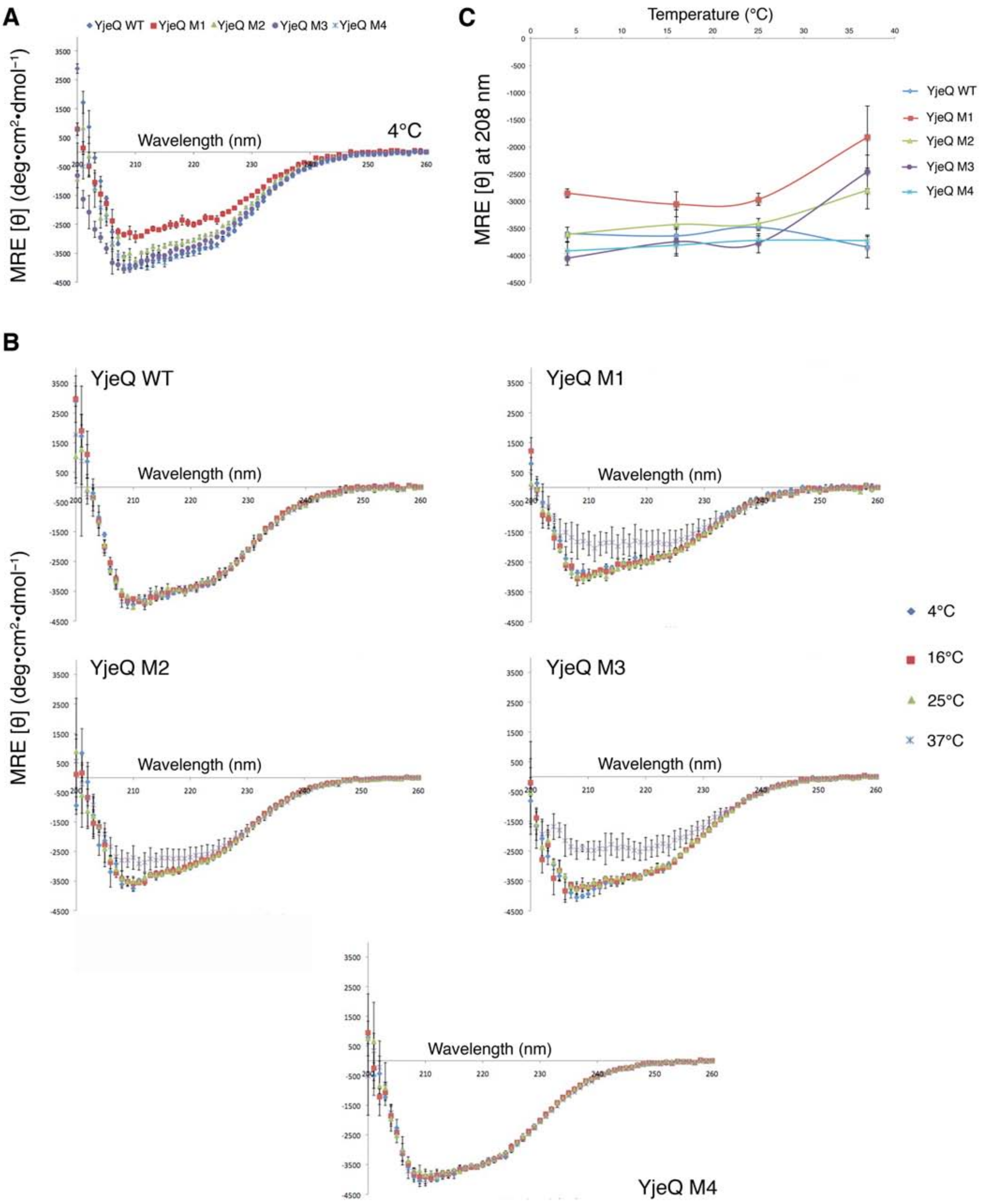
A
YjeQ WT
YjeQ M1
YjeQ M2
YjeQ M3
YjeQ M4
4°C
Wavelength (nm)
MRE [θ] (deg•cm²•dmol⁻¹)
C
Temperature (°C)
MRE [θ] at 208 nm
YjeQ WT
YjeQ M1
YjeQ M2
YjeQ M3
YjeQ M4
B
YjeQ WT
YjeQ M1
YjeQ M2
YjeQ M3
YjeQ M4
Wavelength (nm)
MRE [θ] (deg•cm²•dmol⁻¹)
4°C
16°C
25°C
37°C

Figure S2. Circular dichroism (CD) spectra of YjeQ C-terminal variants. YjeQ, YjeQ M1, YjeQ M2, YjeQ M3 and YjeQ M4 were scanned for 15 minutes in buffer containing high salt (300 mM NH_4Cl) at varying temperatures of 4°C, 16°C, 25°C and 37°C. The CD spectra of each protein were scanned three consecutive times from 200 nm to 260 nm over the incubation time. The mean residue molar ellipticity (MRE) [θ] [deg•cm^2•$dmol^{-1}$] was calculated and plotted to obtain a CD spectra of each protein at the different temperatures. (A) The CD spectra for wild type YjeQ showed a spectrum with negative peaks at 208 and 222 nm, which is consistent with a protein containing both α-helices and β-strands as part of its secondary structure. The spectra for the YjeQ variants showed a similar profile with minimal mean residue molar ellipticity (MRE) values at 208 and 222 nm. However, these peaks were proportionally less pronounced in the YjeQ variants, as a consequence of the removal of some or all of the α-helices in the zinc finger domain by the C-terminal truncations. This was easily observed by plotting the CD spectra of wild type YjeQ and the four YjeQ variants obtained at 4 ºC. The YjeQ M1 variant (with the entire zinc-finger domain being removed) produced the most divergent spectra compared to wild type, whereas YjeQ M2, YjeQ M3 and YjeQ M4 generated a spectrum that almost overlapped with that of the wild type protein. (B) The three scans obtained for each protein at 4 ºC, 16 ºC and 25 ºC showed little variability (left panels). Consequently, the standard deviations obtained for the MRE values of the average spectrum for each YjeQ protein were small. However, at 37 ºC the average spectrum for each of the YjeQ proteins (except the YjeQ wild type and YjeQ M4) showed notoriously higher standard deviation values (left panels) and overall less pronounced negative peaks at 208 and 222 nm. (C) For easy observation of this last feature of the CD spectra we plotted the MRE at 208 nm vs. temperature for wild type YjeQ and the four YjeQ variants. These results indicate that the three YjeQ deletion variants were unstable and partially unfolded during the 15 min incubation at 37 ºC.

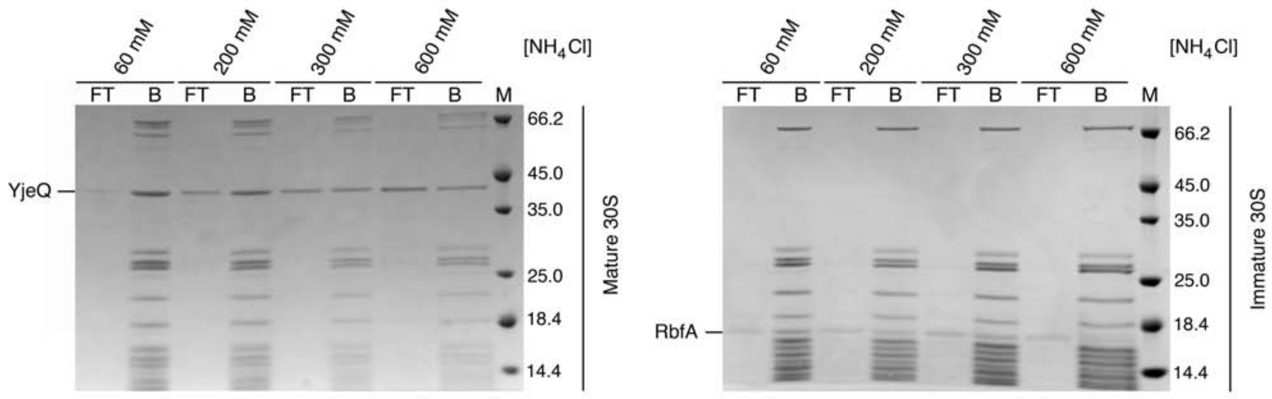

Figure S3. Binding of YjeQ and RbfA to the 30S subunit at different salt concentrations. To establish the NH_4Cl concentration at which binding of YjeQ to the mature 30S subunit and RbfA to the immature 30S subunits becomes stoichiometric, we tested the binding of these factors at different salt concentrations using filtration assays. These particular assembly reactions (YjeQ + mature 30S and RbfA + immature 30S) were chosen because these are the most stable complexes YjeQ and RbfA form. YjeQ and RbfA were added in 5-fold molar excess with respect to the 30S subunits in all reactions. Assembly reactions containing YjeQ were done in the presence of 1 mM GMP-PNP. The unbound protein was captured in the flow-through (FT) and the bound (B) protein was retained in the filter. Fractions were then resolved by 4-12% bis-tris SDS-PAGE and stained with Coomassie blue. Gels were scanned (Biorad ChemiDoc MP Imaging System) and the intensity of the protein bands was measured by densitometry (Image Lab Version 5.0-built 18; Biorad) and compared to the intensity of the bands from YjeQ, RbfA and ribosome standards in the same gel (not shown). Stoichiometric binding of YjeQ to the mature 30S subunit was observed at 300 mM and 60 mM NH_4Cl for YjeQ and RbfA respectively. The molecular weight marker (M) is in kDa.

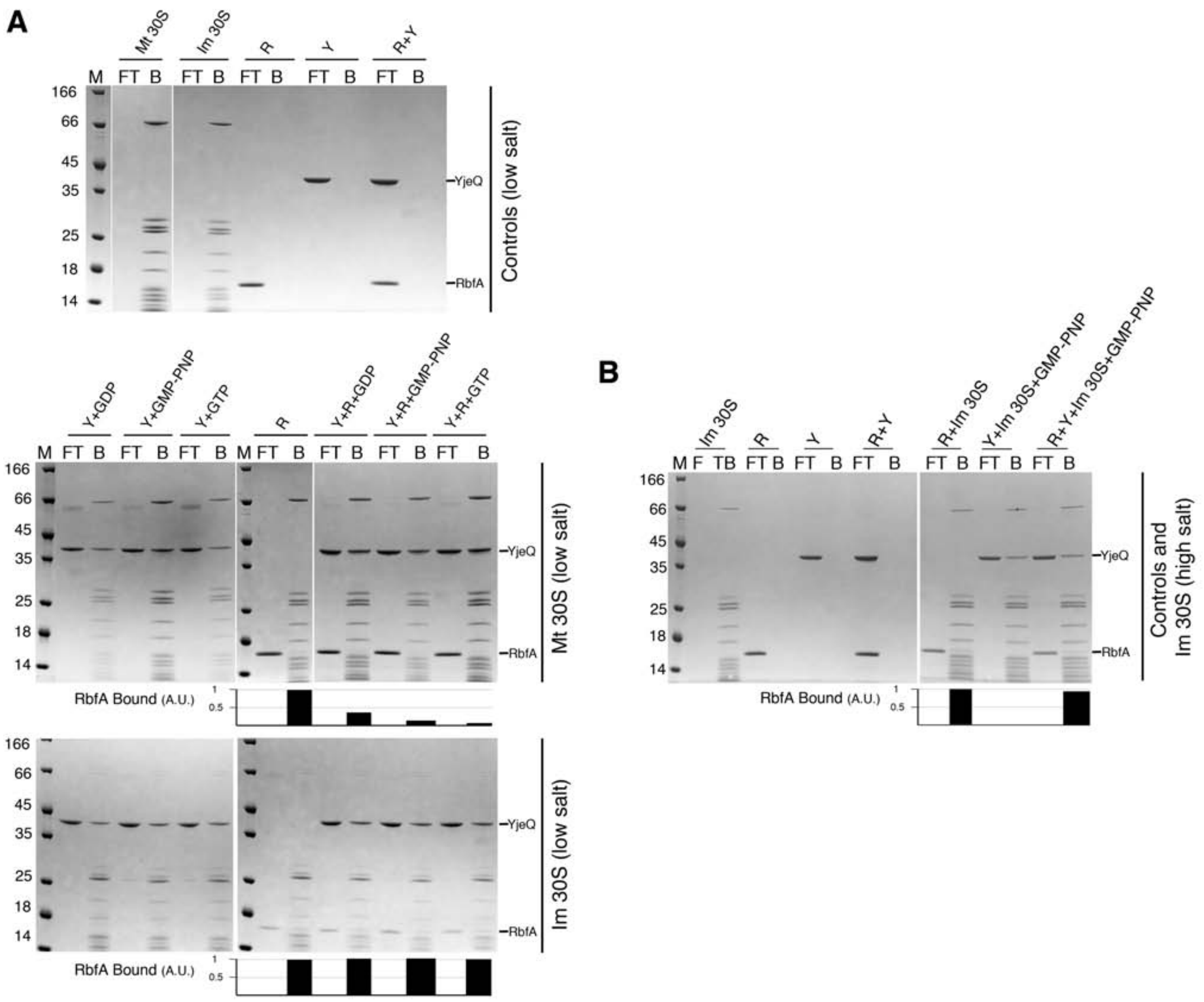

Figure S4. YjeQ removes RbfA bound to the mature 30S subunit. (A) Filtration assays testing the ability of YjeQ (Y) to remove RbfA (R) bound to the mature (Mt) and immature (Im) 30S subunit in buffer containing 60 mM NH_4Cl (low salt). RbfA was first incubated with mature or immature 30S subunits at 37 ºC for 15 min. Then YjeQ and nucleotide (GDP, GMP-PNP or GTP) were added where indicated and the mixtures were incubated for an additional 15 min. Proteins were added in five fold molar excess to 30S particles. Flow-through (FT) and bound (B) fractions containing unbound and bound proteins retained on the filter respectively were separated by ultracentrifugation through a 100-kDa cut-off centrifugal device. Fractions were resolved by 4-12% bis-tris SDS-PAGE and stained with Coomassie blue. The molecular weight for the markers (M) is in kDa. The SDS-PAGE in the top panel shows the control reactions containing YjeQ

(Y), RbfA (R) or the 30S subunits (mature or immature) by themselves. We also tested a reaction containing both YjeQ and RbfA to ensure that combining the two proteins did not cause retention of some protein in the filtration device. (B) The 4-12% bis-tris SDS-PAGE stained with Coomassie blue in this panel shows the experiment in the bottom panel in (A) testing the ability of YjeQ to remove RbfA bound to the immature 30S subunit but performed in buffer containing 300 mM NH_4Cl. The bar diagrams under the gels in (A) and (B) indicate the binding of the RbfA to the mature 30S subunit in each reaction. The observed binding of RbfA to the mature 30S subunit was defined as 1.

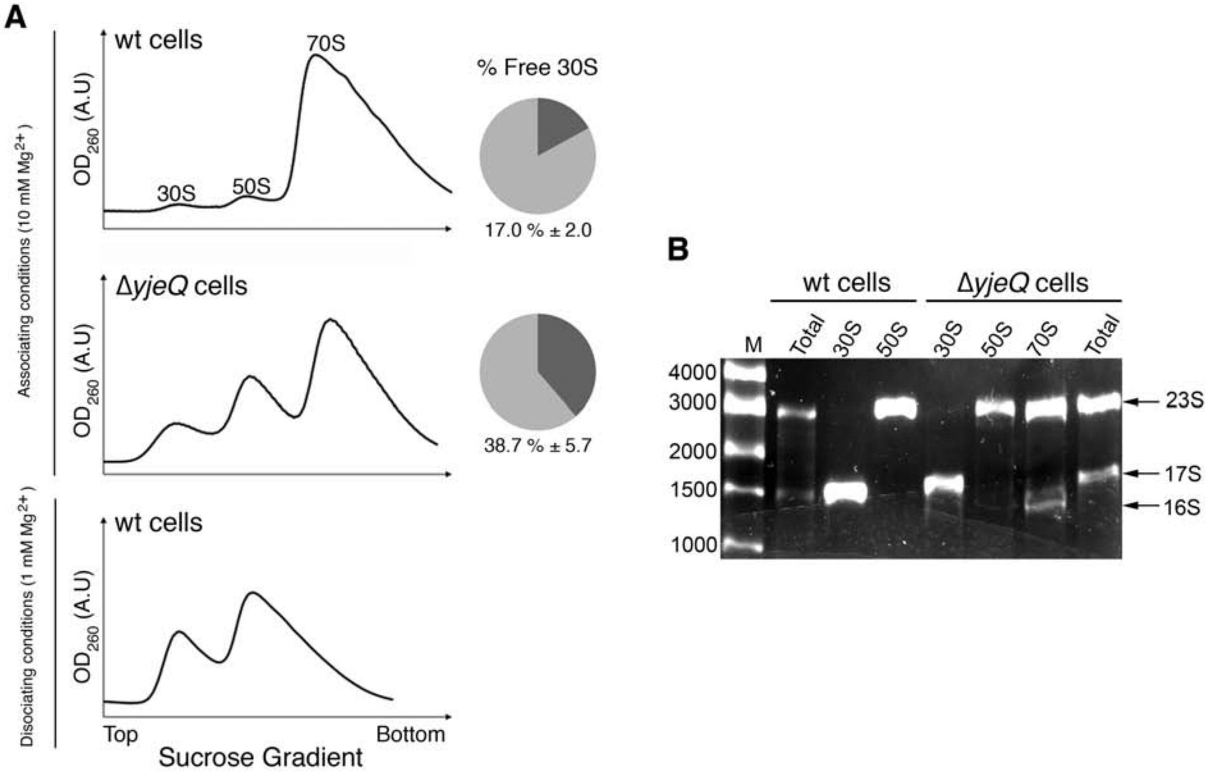

Figure S5. Purification and analysis of the mature and immature 30S particles. (A) Ribosome profiles from parental (wt) and Δ*yjeQ E. coli* cells. The two top gradients were obtained under 'associating conditions' that maintain the two subunits in the 70S ribosome tightly bound. The bottom gradient was generated under 'dissociating conditions' that exposes ribosomes to 1 mM Mg^{2+}concentration and induces the dissociation of the 70S ribosomes, which allow for efficient purification of the mature 30S subunit. The pie charts were calculated from the corresponding ribosome profiles to the left to measure the percentage of free 30S subunits in the cells [peak area for the 30S subunit (free 30S subunits) / total 30S subunits present in the cells (peak area for the 30S subunit + 30% of the area under the 70S peak)]. (B) The total rRNA content from parental and *ΔyjeQ* cells, as well as for the fractions containing the 30S, 50S and 70S particles from the sucrose gradients were resolved in a 0.9% synergel - 0.7% agarose gel. Bands corresponding to the mature 23S and 16S rRNA as well as the immature 17S rRNA are indicated.
